# Immune Checkpoint Inhibitor-Induced Colitis: How Long Does the Threat Last?

**DOI:** 10.7759/cureus.40627

**Published:** 2023-06-19

**Authors:** Paola Michelle Calle Sarmiento

**Affiliations:** 1 Internal Medicine, University of Connecticut, Farmington, USA

**Keywords:** bladder cancer, delayed immune related events, ici colitis, immunotherapy induced colitis, immunotherapy, pembrolizumab

## Abstract

Delayed immune-related events (DIRE) occur after ≥90 days of discontinuation of immunotherapy. Pembrolizumab is a monoclonal antibody that targets the programmed cell death protein 1 (PD-1) receptor of lymphocytes and is used frequently in the management of multiple cancers. Immunotherapy-related adverse events (irAE) are common; most occur six to seven weeks after starting immunotherapy. However, DIRE could also arise months after the stopping therapy. Although many cases of immunotherapy-induced colitis have been reported, data on colitis DIRE is limited. We present the case of a 76-year-old gentleman with bladder cancer who received pembrolizumab and developed significant diarrhea after four months of discontinuation of immunotherapy. His workup included a sigmoidoscopy with a biopsy showing evidence of immune-related colitis. In addition, the patient received steroids achieving complete resolution of diarrhea.

## Introduction

Immune checkpoint inhibitors (ICIs) have become essential in cancer care. Immunotherapy agents enhance immunity against tumor cells by blocking proteins or ligands that generally lead to the inactivation of cytotoxic antitumor T-cells. Programmed death-1/ligand 1 (PD-1/PDL-1) inhibitors and cytotoxic T-lymphocyte-associated protein-4 (CTLA-4) inhibitors have shown significant potential in improving overall survival (OS) in patients with malignant melanoma, non-small cell lung carcinoma, renal cell carcinoma, and other tumors [1]. Immunotherapy-related adverse events (irAE) usually occur during the first 12 weeks after therapy initiation. Diarrhea and colitis typically occur six to seven weeks after starting an ICI [2]. However, irAE can occur much later after initiating or discontinuing therapy, becoming a clinical challenge that needs to be recognized. Delayed immune-related events (DIRE) occur after ≥90 days of discontinuation of immunotherapy [3]. We describe a case of a patient who developed significant diarrhea due to biopsy-proven ICI colitis after four months of discontinuing pembrolizumab used to treat his bladder cancer.

## Case presentation

A 76-year-old male with a history of a T1 non-muscle-invasive urothelial carcinoma of the bladder initially managed with transurethral resection of bladder tumor (TURBT) and Bacillus Calmette-Guerin (BCG) intravesicular chemotherapy. He underwent annual surveillance with cystoscopies and developed a recurrence of his disease. He underwent a second TURBT and BCG induction complicated by BCG cystitis. He was recommended to undergo cystectomy but elected to forgo surgery. Therefore, he was started on the PD-1 inhibitor pembrolizumab. Unfortunately, he could not tolerate pembrolizumab well and developed multiple adverse effects, including profound fatigue, nausea, worsening shortness of breath, and frequent urinary tract infections. Hence, pembrolizumab was discontinued after nine months.

Four months after discontinuing pembrolizumab, the patient presented to the hospital due to profound fatigue decreased mobility and inability to perform activities of daily living (ADLs), and severe non-bloody diarrhea. He reported having 10-12 episodes of watery brown diarrhea a day accompanied by mild diffuse abdominal discomfort alleviated by bowel movements. He denied fevers, chills, cough, shortness of breath, nausea or vomiting, hematochezia, or melena. He denied any recent travel or sick contacts. He had no significant changes in his diet before his presentation. Initially, his vital signs were within normal limits and without fevers. His physical exam was relevant for a frail and ill-appearing elderly male with dry mucous membranes, increased skin turgor, and a soft but mildly tender abdomen diffusely to deep palpation without guarding or rebound tenderness. His laboratory workup showed a white blood cell count of 13.7 K/µL (normal value 4.5-11.0 K/µL), hemoglobin 10.1 g/dL (normal value 12.8-17.0 g/dL), mean corpuscular volume (MCV) of 93.2 fL (normal value 82-99 fL) and platelet count of 251 K/µL (normal value 140-360 K/µL). Chemistries showed an elevated creatinine level of 2.6 mg/dL (normal value 0.5-1.5 mg/dL) from his baseline creatinine of 1.5 mg/dL and electrolyte derangements, including mild hypokalemia and hypomagnesemia. His liver profile was unremarkable.

**Figure 1:**
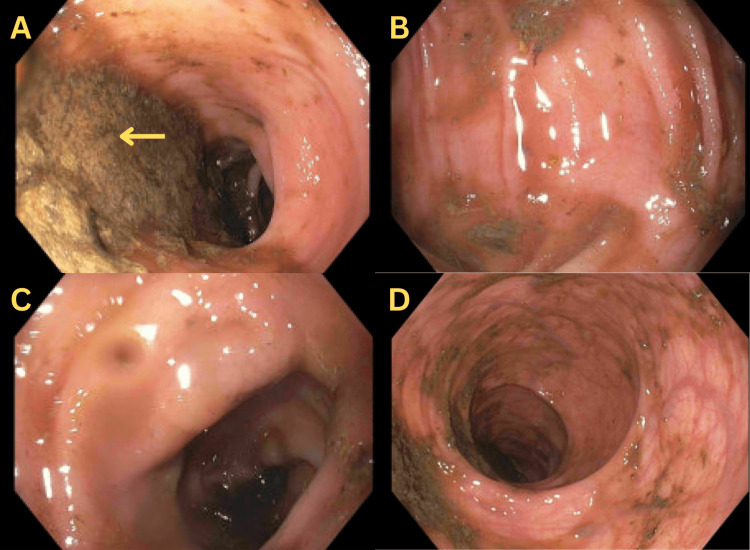
Flexible sigmoidoscopy images showing suboptimal bowel preparation with stool content not allowing full visualization of the colon and rectum (yellow arrow) (A). Nevertheless, the visualized mucosa of the sigmoid colon (B), descending colon (C), and rectum (D) was normal in appearance.

The workup of infectious and medication-induced causes was unrevealing. Therefore, the patient underwent a sigmoidoscopy. Although the preparation was suboptimal, an area of 70 cm from the anus to the descending colon was assessed showing a healthy-appearing mucosa without pseudomembranes (Figures 1A-1D).

**Figure 2:**
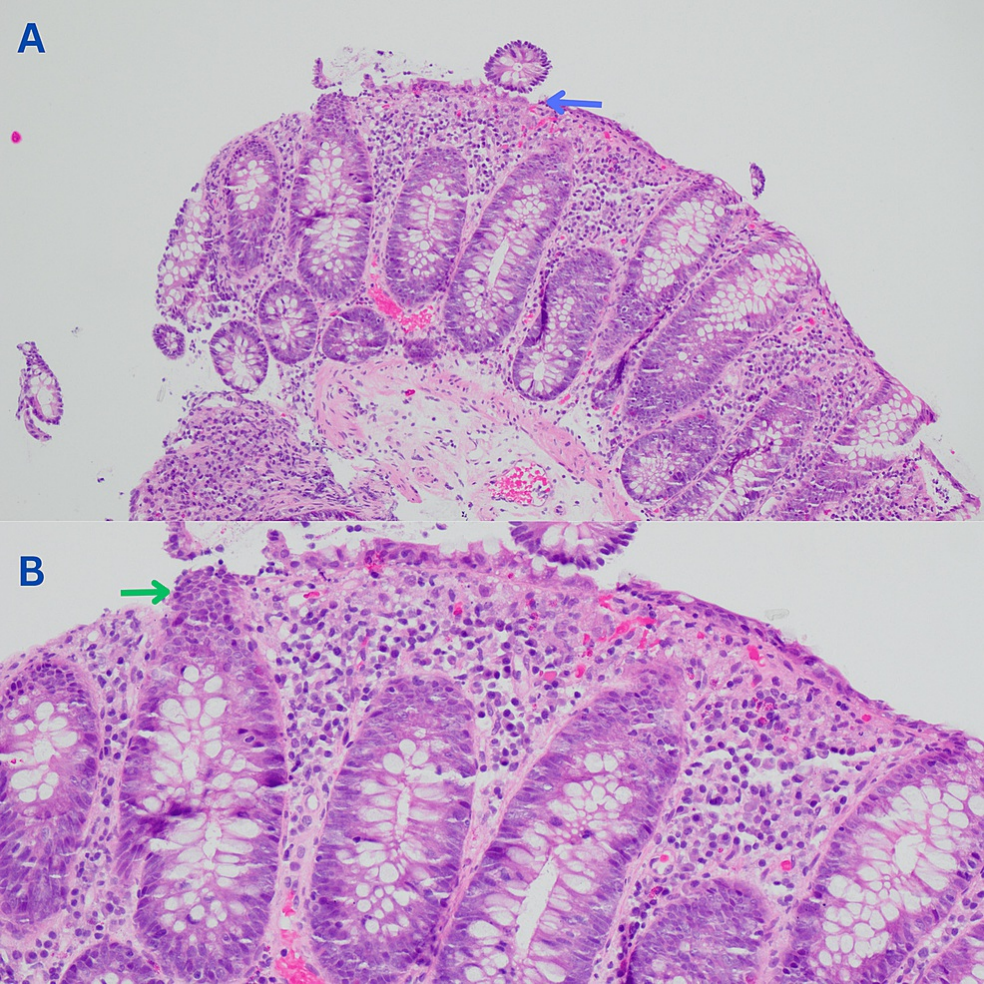
Light microscopy of colonic biopsies Hematoxylin and eosin (H&E) stain showing degenerative changes of the surface epithelium (blue arrow) (A) and increased intraepithelial lymphocytes in the surface of the epithelium (green arrow) (B).

Several random biopsies were taken throughout the colon from descending colon to the rectum showing a colonic mucosa with degenerative changes of the surface epithelium and increased intraepithelial lymphocytes in the surface of the epithelium and crypts. In addition, the lamina propria was expanded with focally increased apoptosis (Figures 2A, 2B). These findings were suggestive of ICI-associated colitis. Therefore, the patient was started on oral prednisone at 1 mg/kg/day with excellent response. His diarrhea improved in the first 72 hours and resolved within a week of starting steroids.

## Discussion

Immunotherapy has revolutionized the field of oncology and cancer treatment. Unfortunately, the use of immunotherapy in cancer management is associated with the development of autoimmune toxicities or irAE. In most cases, irAE occur during the first 12 weeks after therapy initiation [4]. Nevertheless, gastrointestinal adverse reactions such as diarrhea and colitis can occur earlier, typically six to seven weeks after starting an ICI [2]. Some irAE can occur many months after initiating or discontinuing therapy, a phenomenon described as DIRE. DIRE occur after ≥90 days of discontinuation of immunotherapy [3]. Notably, the duration of the threat for DIRE after immunotherapy is still unknown. Long-term side effects with immunotherapy are common. Approximately 43% of patients develop an irAE that lasts for at least three months after finishing immunotherapy [5]. Furthermore, the availability of long-term data is limited since the median duration of safety reporting in clinical trials was 90 days [6]. Thus, case reports and other studies investigating the long-term effects are crucial to better understanding immunotherapy's risks and complications.

The incidence of grade 3/4 diarrhea is higher in patients receiving anti-CTLA-4 agents, such as ipilimumab (10%), compared to patients receiving anti-PD-1/PD-L1 agents, such as nivolumab or pembrolizumab (1%-2%) [7]. Our patient received pembrolizumab for bladder cancer and developed diarrhea 13 months after the start and four months after the discontinuation of therapy. The most important clinical feature of ICI colitis is diarrhea which occurs in approximately 37% of patients [1]. Diarrhea severity can be graded from mild (grade 1) to severe (grade 4). Grade 1 colitis is the increase of fewer than four stools per day over baseline. Grade 2 colitis represents the increase of four to six stools per day over baseline. Grade 3 colitis occurs when there is an increase of seven or more stools per day over the baseline, new stool incontinence, need for hospitalization, and limitations in self-care. Finally, grade 4 colitis occurs when diarrhea causes life-threatening complications requiring urgent interventions [8]. Interestingly, the development of irAEs could indicate benefits in cancer care. Our patient has not developed any signs of recurrence of his disease.

Generally, the diagnostic workup for grade 1 colitis is unnecessary, and diarrhea can be self-limited. However, grade 2 colitis and above requires a workup for alternative causes such as infection, measuring inflammatory markers, imaging, and endoscopic evaluation. Grade 3 and 4 colitis usually requires hospitalization; the workup is needed urgently. Testing can include complete blood count (CBC), comprehensive metabolic panel (CMP), thyroid-stimulating hormone (TSH), erythrocyte sedimentation rate (ESR), C-reactive protein (CRP), and stool (culture, Clostridium difficile, parasites, cytomegalovirus (CMV), other viral infections, and ova and parasites).

Lactoferrin can help stratify patients to determine who needs urgent endoscopic evaluation. In addition, Calprotectin can serve as a marker of disease activity. Other tests required before treatment with infliximab include HIV, hepatitis A and B, and blood quantiferon for tuberculosis. Imaging with computed tomography (CT) scan of the abdomen and pelvis and gastrointestinal endoscopic evaluation are essential since ulceration in the colon can predict a corticosteroid-refractory disease course [7].

The management of grade 1 colitis is primarily conservative and includes bowel rest, adequate fluid and electrolyte replacement, and consideration of temporary delay in ICI treatment. More severe forms of colitis may require treatment with steroids, usually given in a taper over four to six weeks, gastroenterology consultation with esophagogastroduodenoscopy (EGD) and colonoscopy, and other agents such as infliximab or other anti-tumor necrosis factors (anti-TNF therapies) when no response to corticosteroids occurs after 48 to 72 hours [7]. Therefore, the implicated ICI should be discontinued permanently in most grade 3 or 4 colitis scenarios. However, one can consider switching from an anti-CTLA-4 to an anti-PD-1/PDL-1, given the decreased risk of colitis with anti-PD-1/anti-PDL-1 agents [9]. Finally, consider repeat endoscopic evaluation in patients with moderate to severe colitis who do not respond to immunosuppressive agents or disease monitoring when clinically indicated.

## Conclusions

Given the expanding use of ICIs and their increasing role in cancer therapy, it is imperative to enhance our understanding of the duration and persistence of the risk of DIRE. The present case highlights the significance of closely monitoring patients who received pembrolizumab for the development of DIRE, particularly colitis. Furthermore, these complications can manifest after an extended duration of treatment, necessitating ongoing vigilance beyond the initial treatment period. Although isolated case reports provide valuable insights, further data from more extensive studies and registries are needed to assess the temporal dynamics of delayed immune-related adverse reactions comprehensively. Additionally, more data could help develop evidence-based guidelines about the frequency and duration of monitoring required to identify and address potential adverse events promptly.
